# Fabrication and Characterization of Carboxymethyl Starch/Poly(l-Lactide) Acid/β-Tricalcium Phosphate Composite Nanofibers via Electrospinning

**DOI:** 10.3390/polym11091468

**Published:** 2019-09-09

**Authors:** Mohd Reusmaazran Yusof, Roslinda Shamsudin, Sarani Zakaria, Muhammad Azmi Abdul Hamid, Fatma Yalcinkaya, Yusof Abdullah, Norzita Yacob

**Affiliations:** 1 Faculty of Sciences and Technology, National University of Malaysia, Bandar Baru Bangi 43600, Selangor; 2 Institute for Nanomaterials, Advanced Technology and Innovation, Technical University of Liberec, 46117 Liberec, Czech Republic; 3 Material Technology Group, Malaysian Nuclear Agency, Bangi, Kajang 43300, Selangor

**Keywords:** poly l-lactide (PLLA), carboxymethyl starch (CMS), β-tricalcium phosphate (β-TCP), electrospinning, nanofibers

## Abstract

A natural polymer of carboxymethyl starch (CMS) was used in combination with the inorganic mineral of β-Tricalcium Phosphate (β-TCP) and Poly l-lactide (PLLA) to prepare composite nanofibers with the potential to be used as a biomedical membrane. β-TCP contents varied in the range of 0.25% to 1% in the composition of PLLA and CMS. A mixed composition of these organic and inorganic materials was electro-spun to produce composite nanofibers. Morphological investigation indicated that smooth and uniform nanofibers could be produced via this technique. The average of the nanofiber diameters was slightly increased from 190 to 265 nm with the β-TCP content but some agglomeration of particles began to impede in the fiber at a higher content of β-TCP. It was observed that the fibers were damaged at a higher content of β-TCP nanoparticles. With the presence of higher β-TCP, the wettability of the PLLA was also improved, as indicated by the water contact angle measurement from 127.3° to 118°. The crystallization in the composite decreased, as shown in the changes in glass transition (*T*_g_) and melting temperature (*T*_m_) by differential scanning calorimeter (DSC) and X-ray diffraction analysis. Increases in β-TCP contributed to weaker mechanical strength, from 8.5 to 5.7 MPa, due to imperfect fiber structure.

## 1. Introduction

There are numerous techniques to fabricate nanofibers, including drawing, phase separations, template synthesis, self-assembly, and electrospinning [1,2,3,4]. In comparison, electrospinning, which has become an interest of a great many researchers, is a cost-effective and easy to control process which produces uniform nanofibers. Nanofibers produced by electrospinning provide a huge surface area to volume ratio, high porosity, and sufficient mechanical characteristics [5]. The distribution of fibers diameter and porosity is easy to control by controlling the processing parameters of electrospinning. This versatile technique is suitable for the fabrication of various formulations of nanofibers usable in multiple applications including filtration [6,7,8,9,10,11], drug delivery systems [12,13,14], the textile industry [15,16], or scaffolds for tissue engineering [17,18,19,20]. In biomedical engineering areas, nanofibers prepared via electrospinning provide valuable properties since the nanofibers mimic the extracellular matrix (ECM) which is important as a cell platform. Electrospinning involves three major components—a high voltage, a collector, and a syringe pump. High voltage provides an electrical field between the nozzle and the collector. A syringe pump pushes the polymer solution to the nozzle and controls the flow rate, whereas nanofibers are deposited onto the collector [21]. When the high voltage is applied, an electrical field is generated between the nozzle and the collector, the polymer solution is then electrically charged and drawn to the polymer jet due to a high potential. The polymer jet then stretches and forms fibers. Due to the repulsive forces existing in the polymer jet, the jet becomes unstable and lashes in circular motion while splitting into smaller fibers before being deposited onto the collector.

Presently, numerous types of composite nanofibers have been developed, such as synthetic–synthetic polymers [22,23], natural–synthetic polymers [24,25,26,27] and natural–synthetic–particulate nanofibers [28,29,30,31]. Natural polymers have become a great interest of many researchers due to a number of advantages such environmental friendliness, low costs, and easy extraction from a natural resources that are available in many countries. In industrial applications, a natural polymer of carboxycellulose nanofibers derived from jute has exhibited a very high mechanical strength compared to the raw jute fibers in nanopaper applications [32]. Carboxycellulose nanofibers produced by the nitro-oxidation method have been applied as a uranium oxide remover from water. The nanofibers exhibit a higher high surface charge and large carboxylate content allowing them to be used as an effective medium to remove UO_2_^2+^ ions from water [33]. Sharma et al. 2018 [34] derived that the nanocellulose from spinifex can serve as an absorbent material. The nanocellulose fibers successfully absorbed heavy metal like cadmium (II) in water. 

In improving the properties, the nanofibers are not relying on a single-phase polymer but can be mixed with other polymers or composed with inorganic mineral. In the case of tissue engineering applications, cell interaction has become a major factor to scaffolds beside the structural integrity. Besides the blend between synthetic to synthetic polymers, natural polymers are starting to get greater attention as some synthetic polymers are hydrophobic. Natural polymers are becoming an alternative to improve the hydrophilicity of the scaffold. However, natural polymers have no structural integrity in their nature and are not spinnable to the nanofiber form. Ming He [35] has produced feather keratin/PVA composite nanofibers with a fiber diameter distribution between 200 to 500 nm. Increasing natural polymer contents led to a reduction in fiber diameter. Tensile strength was observed to decrease with the higher content of feather keratin. The application of hydroxyethyl cellulose and collagen improved in vitro biodegradation after being composed with PVA. Mechanical strength was measured slightly higher with the presence of collagen [36]. Hydroxyethylcellulose (HEC) was used to compose polyvinyl alcohol (PVA) and collagen to produce a nanofiber membrane for tissue engineering applications. The HEC/PVA/Collagen exhibited good degradation properties that met the requirements in dermal replacement [37]. Carboxymethyl cellulose was used with poly(ethylene oxide) and successfully produced the core-shell nanofibers for use in drug-release systems. The nanofibers showed excellent bactericidal activity against a wide range of bacteria, indicating their potential use as antibacterial materials in various applications such as tissue engineering and pharmaceutical science [38]. Gelatine as protein sources has been used in composition with polycaprolactone (PCL) followed by crosslinking with genepin for guided bone regeneration (GBR). Increased gelatine content in PCL increased the wettability and cell adhesion of the nanofibers, but the elasticity of the composite nanofibers was observed to have been reduced [39]. 

In GBR applications, a number of natural polymers have being used with inorganic materials such as collagen and hydroxyapatite (HA). The combination of polymer with HA in nanofiber fabrication has shown good bioresorbability and osteoconductivity but low toughness and plasticity [40]. Composite nanofibers of gelatin–HA were successfully electrospun and the produced diameter of the nanofibers were in the range of 200–400 nm with the highest mechanical strength of approximately 4.7 MPa. Nanofibers were indicated to have a good cell adhesion that is suitable for GBR [41]. Song et al. 2014 [42] electrospun PLLA grafted with HA particles for GBR applications. The produced fiber diameters were approximately 300–500 nm with mechanical strength in the range of 3.5 to 1.5 MPa, depending on the particle content. The water content angle was reduced to 62° at the higher HA content. The osteoblast cells were well adhered and spread over the membrane, indicating a potential for GBR applications. 

**Figure 1:**
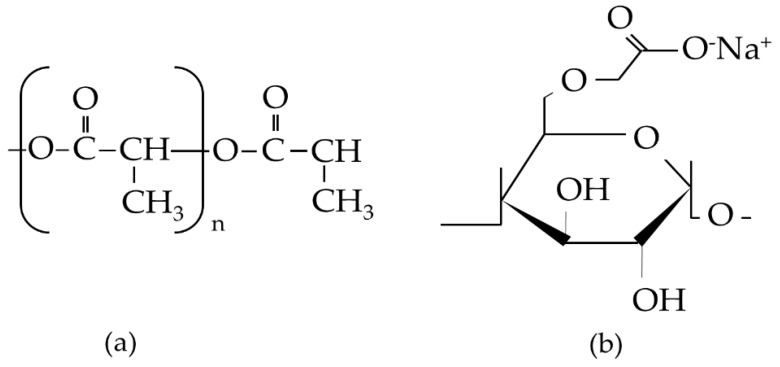
Formula structure of (**a**) poly(l-lactide acid) (PLLA) and (**b**) carboxymethyl starch (CMS).

In this work, we used the natural polymer of carboxymethyl starch (CMS) derived from local sago starch with PLLA and the inorganic mineral of β-TCP. The effect of CMS on the PLLA was reported in our previous work [43]. CMS was used as a polysaccharide source that had undergone the carboxymethylation process from the sago starch by having the O–H group in starch substituted with the ether group in the presence of sodium monochloroacetate in a strongly alkaline environment. PLLA is known to be biocompatible and has been used in various biomedical applications for many years. A formula structure of PLLA and CMS is described in Figure 1. β-TCP was used in a different area of bone regeneration due to its excellence in biocompatibility and degradation rates. Herein, both organic and inorganic components were combined in the form of nanofibers with the potential to be used in the biomedical field. Such a study, to the best of our knowledge, is the first of its kind and may, therefore, lead to the development of GBR.

## 2. Materials and Methods

Poly(l-lactide acid) with the inherent viscosity of 2.32 dL/g was obtained from BioInvigor (Taipei, Taiwan). β-TCP nanopowder was purchased from Berkeley Advanced Biomaterials Inc. (Berkeley, CA, USA), with an average particle size of approximately 250 nm. Carboxymethyl starch was prepared from local sago starch. Ten grams of sago starch was stirred in 300 mL isopropanol (Merck, GmbH, Germany) with an addition of 30 wt % NaOH in a reactor flask equipped with a reflux condenser and burette. In this work, the carboxymethylation process of sago starch was prepared as described by Yaacob et al. [44]. Poly(l-lactide acid) solutions with 7 wt % concentration were prepared by dissolving the granule PLLA in dichloromethane by stirring the mixture for 24 h using the magnetic stirrer. CMS solution was prepared at 10 wt % concentration and then mixed with PLLA in 5% *v/v* of CMS content in PLLA of a total of 5 mL PLLA/CMS solution. A different ratio of β-TCP of 0.25. 0.5, 0.75, and 1 wt % were mixed to PLLA/CMS solutions. Sodium dodecyl sulfate (0.2 wt %) was added to prepare PLLA/CMS solutions to improve the homogeneity of the mixtures. The samples were ultrasonically dispersed, stirred for 72 h, and rotate in circular motion to homogenize the mixture.

### 2.1. Electrospinning

**Figure 2:**
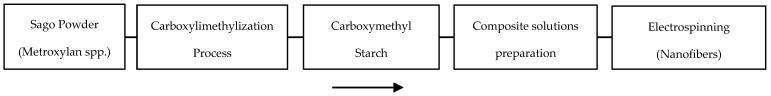
Processing route from a sago powder to electrospinning process to obtain the composite nanofibers.

The composite PLLA/CMS/β-TCP polymer solution was placed into a 1 mL syringe with a 0.6 mm diameter of a blunt needle tip. Distance between the needle and collector was set to 12 cm and connected to a high voltage source (Gamma High Voltage Research Inc., Ormond, ES40P, 20 W, Ormond Beach, FL, USA). Voltage was set to 10 kV, and the syringe pump (New Era Pump System Inc. NE 1000, Farmingdale, NY, USA) was placed vertically. The flow rate of the polymer jet was set to 0.006 mL/min, and the nanofiber mat was collected on an aluminum foil. The surface of fibers was characterized by using a scanning electron microscope (SEM, Quanta 400, FEI, Hillsboro, OR, USA). The images were collected at ×2000 and ×10,000 magnification at 10 kV. The fiber diameter and surface texture were analyzed and calculated by using an image J and StatGraphic Plus software (Statgraphics Technologies Inc**.,** The Plains, VA, USA). The fabrication routes from the sago to the composite nanofibers are summarized in Figure 2.

### 2.2. Characterizations

#### 2.2.1. Chemical Interactions

The chemical interaction analysis was performed by using Fourier transform infrared (FTIR) spectroscopy (Perkin Elmer, Waltham, MA, USA). The FTIR spectrum was measured in the spectral range of 400 to 4000 cm^−1^, which was performed at 16 scans per sample.

#### 2.2.2. Thermal Behavior

Changes in thermal properties were investigated by using the differential scanning calorimeter (DSC, TA Instrument, Q20, New Castle, DE, USA). About 2.35 mg samples were heated from 20 to 250 °C at a heating rate of 10 °C/min. Approximately 35 mL min^−1^ of nitrogen gas was blown into the sample to avoid the oxidation during heating.

#### 2.2.3. X-ray Diffractions (XRD)

The crystallinity and amorphous regions were investigated using an XRD instrument (Bruker, AXS D8, Bremen, German). Samples were scan from 5° to 60° of 2θ angle with a step size of 0.02°/sec. The X-ray source was from the CuKα with the wavelength of 1.5140. The Xpert High Score Plus software was used to analyze the XRD. Fitting of the XRD curves was carried out using the Gaussian equation by Gnuplot software to determine the full wave half maximum (FWHM). The Debye–Scherrer equations were applied to determine the changes in crystallite size in samples.

#### 2.2.4. Wettability

Wettability of the nanofiber surfaces was determined by water contact angle (WCA, One Attention Theta, TL100, Biolin Scientific, Espoo, Finland). 2 μL of distilled water was dropped on the surface of the nanofibers. 130 of WCA data points were collected in 12 s.

#### 2.2.5. Mechanical Properties

**Figure 3:**
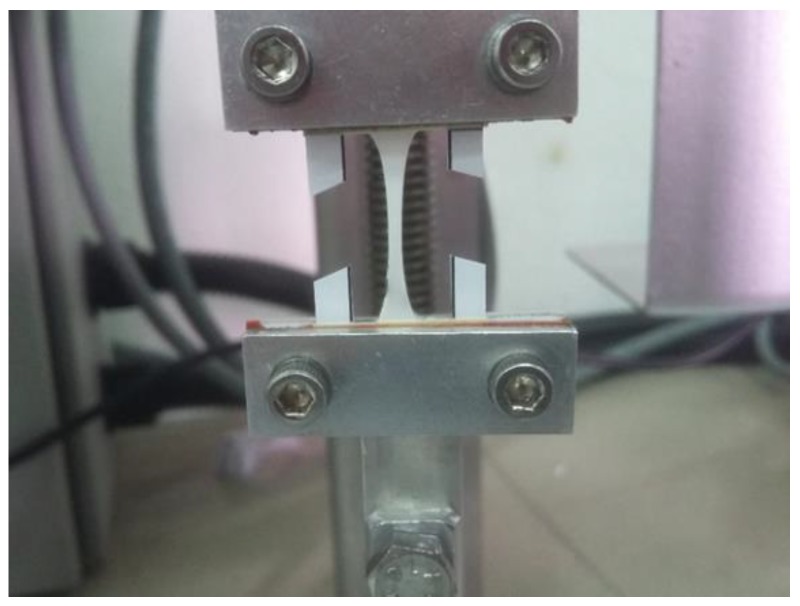
Set up of sample position in tensile test.

The composite PLLA/CMS/β-TCP nanofibers were formed into a rectangular shape (5 × 15 mm) with 10 mm of the gauge length for a tensile test. Samples were removed from the aluminum foil using a paper frame with double-sided tape attached to it. The frame provided additional support to the sample for handling during the testing process (Figure 3). Tensile testing was conducted by using 20 N load cell (Model UUK 5, Chungcheongbuk-do, Korea) equipped with a micro-stepper motor system (Ezi Step, Fastec, Bucheon, Korea) and an OMRON RXRX25 data logger to record the load. The elongation was determined by a 1.0 mW Omron laser detector with a detection limit of 2.5 ms/600 nm. The tensile test was conducted at 0.5 mm min^−1^ of the crosshead velocity. The tensile strength was taken as the maximum stress of the stress–strain curve.

## 3. Results and Discussion

### 3.1. Morphology of PLLA/CMS/β-TCP Composite Nanofibers

**Figure 4:**
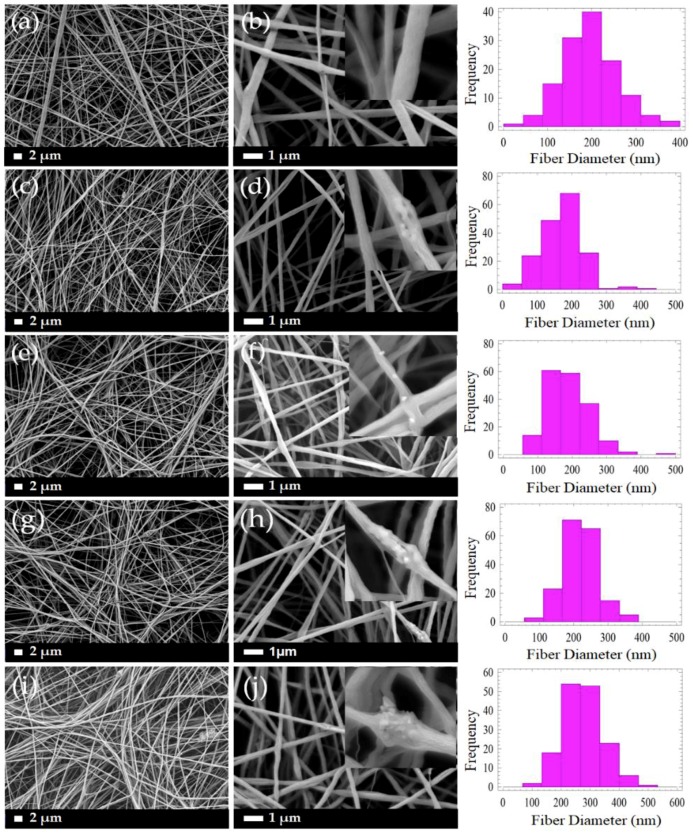
Morphology of the PLLA/CMS/β-TCP composite nanofibers with different compositions of β-TCP at different magnifications of 2000× and 10,000× (**a**,**b**) PLLA/CMS (**c**,**d**) 0.25% β-TCP, (**e**,**f**) 0.5% β-TCP, (**g**,**h**) 0.75% β-TCP and (**i**,**j**) 1% β-TCP in PLLA/CMS.

**Figure 5:**
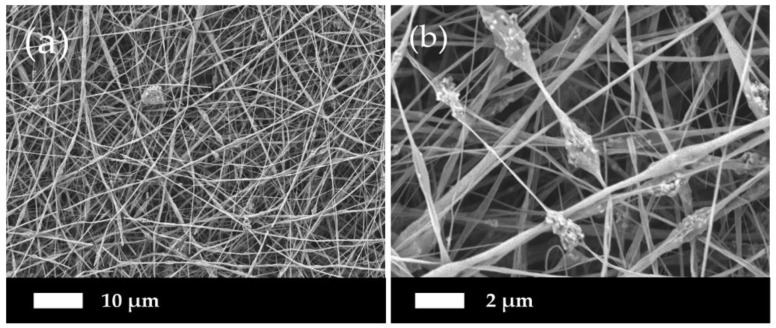
Higher content of β-TCP (6%) particles damaging the structure of nanofibers at magnification of (**a**) 1000× and (**b**) 5000×.

Figure 4 demonstrates the micrographs of the composite PLLA/CMS/β-TCP with different concentrations of the β-TCP particles in the nanofibers. A low content of β-TCP (0.25% to 1 wt %) was added to avoid possible fiber breaking during the electrospinning. It can be observed that smooth, beadless, homogenous, continuous, and randomly oriented nanofibers were obtained. A highly porous and interconnected pore structure were also obtained via this method. A cluster was observed in the nanofibers with the presence of β-TCP particles indicating that a combination interaction had occurred between the particles. As a comparison in morphology observations, the addition of 6 wt % of β-TCP was found to deface the nanofibers structure due to the higher content of the particles in the polymer structure, as indicated in Figure 5. The presence of a higher content of the particles led to the agglomeration in the nanofibers. This agglomeration defaces the formation of nanofibers due to the stretching of the jet polymer during the electrospinning process and results in the discontinuation and breakage of nanofibers. Agglomeration also hinders the movement of jet polymers and the formation of the Taylor cone at the nozzle which leads to non-uniform nanofibers. The agglomeration that formed clusters in nanofibers has been found at a content as low as 0.75 wt % of β-TCP.

**Figure 6:**
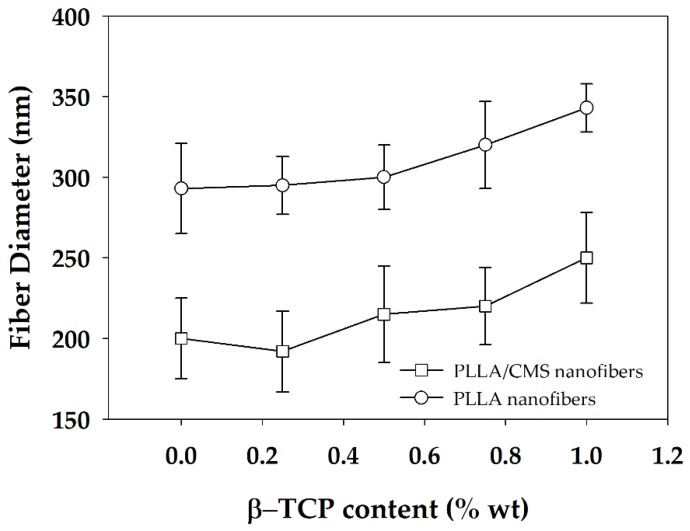
Average diameter of PLLA/β-TCP and PLLA/CMS/β-TCP nanofibers at the different compositions of β-TCP.

The distribution of nanofiber diameter generally changed in the range of 20 to 400 nm for PLLA/CMS with the content of 0.25 to 0.75 wt % of β-TCP. The diameter distribution was increased to approximately 500 nm when the concentration was raised to 1 wt %. The distribution was shifted to the right, as shown, indicating that the diameter of nanofibers was increased with the increase in the composition of β-TCP particles. The increased formation of the cluster due to agglomeration contributes to this phenomenon. Figure 6 demonstrates the average diameters of the PLLA/β-TCP and PLLA/CMS/β-TCP composite nanofibers with the concentration of the β-TCP ranging from 0.25% to 1%. A similar increasing trend was observed with and without the natural polymer of CMS. Siqueira et al. [45] observed a similar trend for the composite PLLA/β-TCP with the composition of β-TCP in the range between 1% to 8%. The average of the nanofiber diameters increased from 260 to 460 nm with the increasing particle content due to the cluster forms in nanofiber structure. The other possible reason that contributes to the increase in diameter is the alteration in the viscosity of the polymer solutions after the addition of β-TCP particles. A higher surface area absorbs liquid during the mixing process and increase the viscosity of the polymers solutions. An increase in viscosity reduces the stretching of the polymer jets during electrospinning and leads to increases in the diameter of the fibers [43,46]. However, the reduction of fiber diameter with the increase of amorphous calcium phosphate (ACP) particles in PDLA fibers was reported by Ma et al. [30] at a certain level of the composition. The increasing of ACP particles to higher composition was found to have no significant effect on fiber diameter. In his report, Ma did not include further discussion on this phenomenon. The breakage, discontinuity, and rough surface of PLLA/HA nanofibers were also observed by Zhao et al. [28]. This effect is more intense at a higher concentration of HA particles. However, Zhao also found out that there was no significant effect on the diameters with the changes in the concentration of HA particles. In our case, we suggest a higher polarity between PLLA/CMS and β-TCP influences the agglomeration effect and the weakness interface bonding between the two.

### 3.2. FTIR Analysis

**Figure 7:**
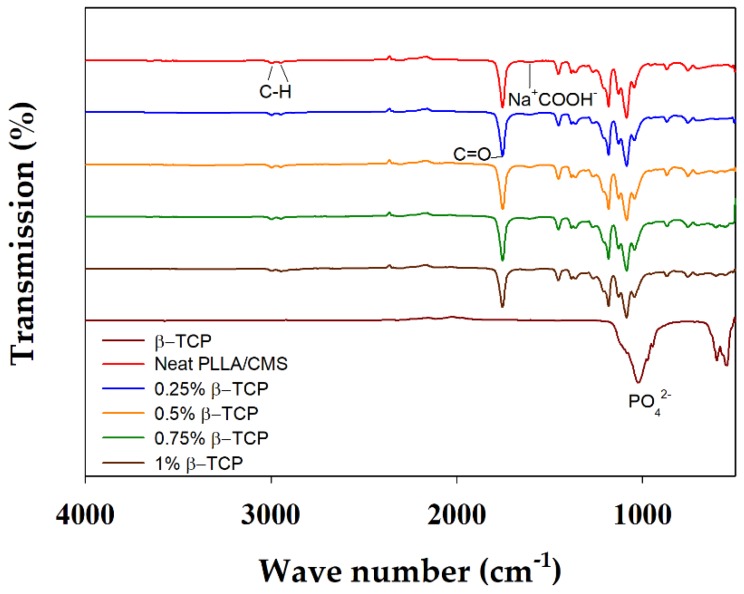
IR Spectrum of composite nanofibers with the different concentration of β-TCP.

**Figure 8:**
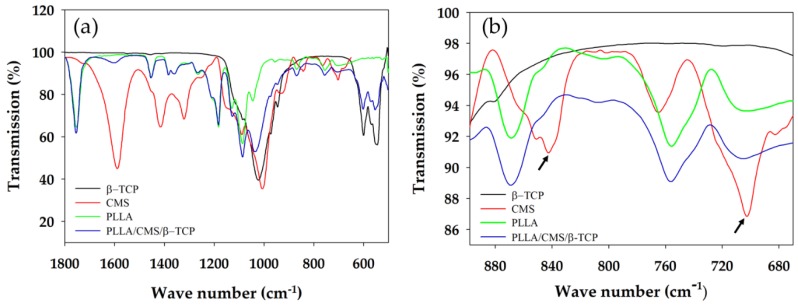
IR absorption peak of composite nanofibers (**a**) Intensity changes and budging and (**b**) peak vanishing (arrow) at the CMS spectrum after the addition of β-TCP particles.

Chemical interactions between the matrix and other component of the composite nanofibers were analyzed by FTIR, as indicated in Figure 7. β-TCP can be characterized by the absorption bands of 946 and 1023 cm^−1^ that rises from the stretching of symmetry and anti-symmetry of P–O bonding, respectively. The O–P–O bond was indicated at 601 and 547 cm^−1^ of the absorption band. The low intensity peak of 1085 cm^−1^ was rising from the portion of crystal structure in the amorphous region [47]. The absorption band of the CMS hydroxyl group appeared at 3200–3400 and 1550–1660 cm^−1^, and was attributed to the COO^−^Na^+^; whereas the absorption peaks of 2995 and to 2945 cm^−1^ corresponded to C–H in PLLA. Peaks at 1767, 1453, and 1383 attributed to C=O presence in PLLA. The peak of 1023 cm^−1^ was observed to budge at 1036 cm^−1^ after the addition of β-TCP into PLLA/CMS (Figure 8a). A possible reason is that it may have resulted from the interface complexes bonding with a hydroxyl group in CMS via oxygen atoms. A similar reaction was observed by Chen et al. [48], a formation of complexes bonds on the surface by the cation transfer between OH groups in CMS and O group in alumina. This condition has resulted in the budging and widening of the FTIR spectroscopy absorption band. The peaks of 839 and 702 cm^−1^ corresponding to CMS structure were found to vanish, which is attributed to the changes in C–C bond in the polymer network of CMS (Figure 8b). The intensity of the carboxymethyl group, CH_2_COO^−^Na^+^ at 1600 cm^−1^, was significantly reduced after the concentration of β-TCP was increased. The difference in intensity corresponds to the reduction of the CH_2_ COO^−^Na^+^ by calcium atoms. β-TCP tends to interact with CMS structure compared to the PLLA, but no significant change for the PLLA IR spectrum was observed.

### 3.3. Differential Scanning Calorimeter (DSC)

**Figure 9:**
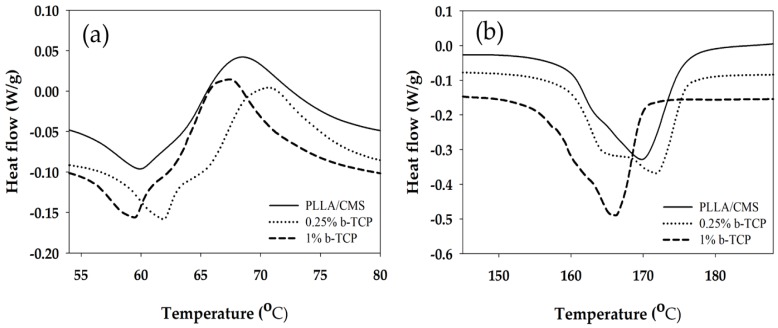
Differential scanning calorimeter **(**DSC) curves show the (**a**) glass transition temperature (*T*_g_) and (**b**) *T*m for PLLA/CMS/β-TCP composite nanofibers at different concentration of β-TCP.

Changes in composite nanofibers properties with regards to temperature were analyzed using DSC room temperature up to 250 °C. The sample without β-TCP, low and high composition of β-TCP particles were analyzed to investigate the influence of β-TCP on the composite nanofibers. Figure 9a depicts the curves of the glass transition (*T*_g_) and cold crystallization (*T*_cc_) temperatures for composite nanofibers for two different compositions of β-TCP.

*T*_g_ increased from 53 to 56 °C after 0.25 wt % of β-TCP was added to PLLA/CMS nanofibers. A slight increase in *T*_g_ indicated the movement of macromolecules of the polymer chain which was obstructed by the particles during heating. There was no significant change in *T*_g_ after more of β-TCP was added to the mixture to up to 1%, suggesting that the damage in the polymer structure due the particles increases and eases the movement of polymer chains. Gay et al. [49] observed a similar condition in PLLA/HA composite nanofibers. The *T*_g_ was reported to increase after HA particles were added to PLLA nanofibers. However, there were no significant changes in *T*_g_ observed by Siquiera et al. [45] with the addition of β-TCP particles in PLLA nanofibers while Ferri et al. [50] reported a slight decrease in *T*_g_ values when β-TCP particles were added in PLLA nanofibers that related to semi hydrolysis of polymer chains with the present of β-TCP. 

Melting temperature (*T*_m_) decreased from 170 to 166 °C at the concentration of 1% of β-TCP, while there were no significant changes at lower concentrations (0.25 wt %) (Figure 9b). Changes in *T*m can be related to the changes in crystallization. The imperfection of crystallization present in nanofibers may contribute to the decrease in melting temperature. A degree of crystallization, X_c_ (Table 1), was found to decrease with an increase of β-TCP concentration. The degree of crystallization reduced from 38.2% to 22.0% after an increase to 1 wt % concentration. This observation can be related to β-TCP that did not react as a nucleation agent and hindered the micro molecule by infiltrating the growth of the crystal. A similar observation was also reported by Siqueira et al. [45], where β-TCP did not act as a nucleation agent to increase the crystallization in the sample. However, low crystallization contributes to better biodegradation by increasing a degradation rate of PLLA in vitro and vivo [51,52]. On the other hand, Ferri et al. [50] observed the increase in *T*m melting temperature with the addition of β-TCP in PLLA due to the presence of spherulite crystal structure and d-lactide acid which does not contribute to the to β-TCP particle crystal growth. However, Hu et al. [53], summarized that β-TCP acts as a nucleation agent as the degree of crystallization was found to increase in PLLA with the addition of β-TCP based on the classical theory of nucleation. A similar observation was reported by [54] during the application of HA particles in PLLA.

### 3.4. X-ray Diffraction Analysis (XRD)

**Figure 10:**
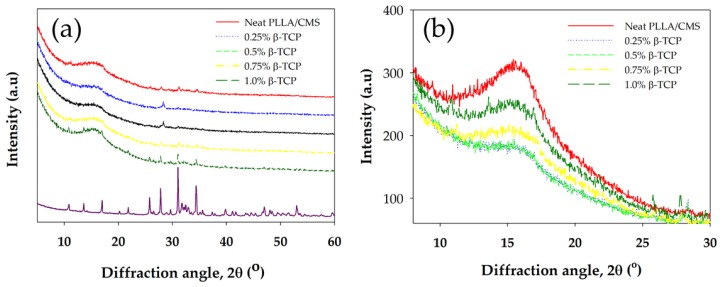
(**a**) X-ray diffraction spectrum for PLLA/CMS/β-TCP composite nanofibers at the different components of β-TCP (**b**) Different intensity of XRD peak at [110] PLLA plane.

X-ray diffractions spectrum (XRD) of PLLA/CMS/β-TCP composite nanofibers are depicted in Figure 10a,b with a scanning angle ranging from 5° to 60° and 8 to 30°of 2θ. PLLA can be characterized by peaks of 15.2° and 19.0° for plane [110] and [203] of the crystallization peaks. CMS did not show crystallization behavior, indicating the crystal structure has become damaged during the modification process from starch to CMS. Peaks of 27.9, 31.1, and 34.3° indicated the primary peak of crystallization of β-TCP for the primary plane being 214, 300, and 220. Peaks of PLLA fibers were observed to be wider compared to the granules PLLA after the electrospinning process, which would suggest it was due to the stretching of the liquid polymer coupled with rapid solidification. Generally, the XRD spectrum of the composite nanofibers demonstrates a wide peak and this indicates that amorphous structure exists in the composite. The intensity of the PLLA peaks was slightly decreased with the increase of the β-TCP due to the decrease in the degree of crystallization, as indicated in DSC data (Table 1). The peak of 0.25% β-TCP has not appeared in the spectrum, which may be due to too low a concentration in the composite to be detected. The intensity of the peak of the composite nanofibers at 13.5° and 17.0° increased with the increase in the β-TCP content. The peak in the spectrum is contributed to the amount of β-TCP that existed at closer peak to PLLA and is not contributed by polymer crystallization (Figure 9b). The decrease in intensity is also due to combinations apart of amorphous regions of inorganic material in nanofibers [55].

**Figure 11:**
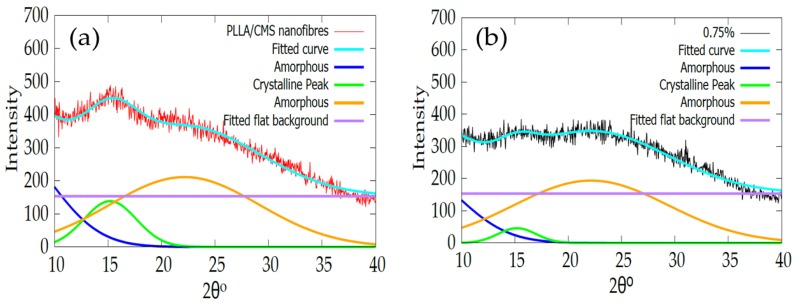
Indicates the changes in crystallinity peaks of 2 different compositions of (**a**) without β-TCP and (**b**) 0.75% β-TCP fitted with the Gaussian equation.

The intensity of the XRD peak of PLLA/HA nanofibers also exhibited a similar pattern after the addition of the HA particle via electrospinning [56]. Kim et al. [41] also found a reduction in intensity and peak widening after calcium phosphate was added to create a gelatin of the nanofibers solution. The possible reason is due to low crystallinity or changes in crystal size to a smaller crystal. Table 2 shows the change in crystallite size with the increase in β-TCP content in the composite nanofiber. The increase in β-TCP in the sample resulted in a decrease in crystallite sizes. The sample with a batch of β-TCP exhibited lower peak intensity and FWHM according to the data compared to the sample without of β-TCP particles. However, at 1% of β-TCP, the crystallite size cannot be obtained. This phenomenon may due to inference with the crystallinity peak of the β-TCP mineral at a high amount (1%). Figure 11a,b show the fitting curves of crystallinity change of the composite with and without the β-TCP particle.

### 3.5. Wettability

**Figure 12:**
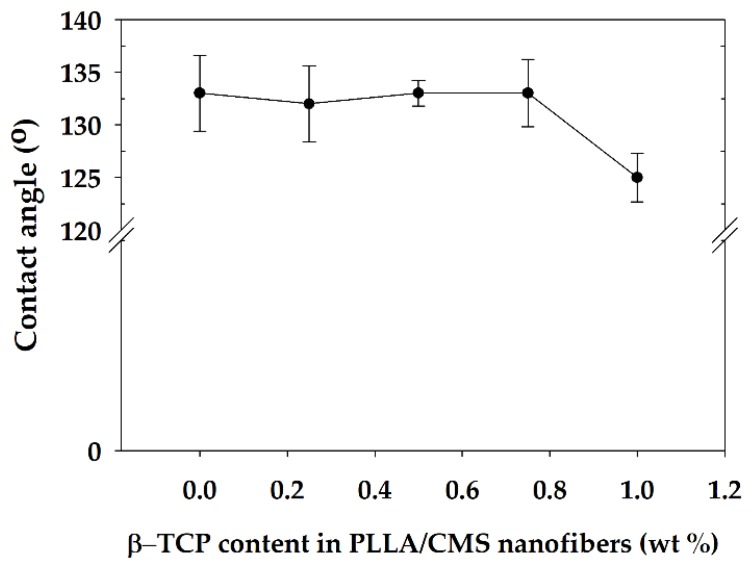
Water contact angle (WCA) of PLLA/CMS nanofibers at different contents of β-TCP.

The hydrophilic and hydrophobic properties of the composite nanofibers were analyzed by the water contact angle (WCA). Several studies have indicated that a hydrophilic surface encourages cell adhesion compared to a hydrophobic surface [57]. There was no significant change in WCA for composition 0.25 to 0.75 wt % except for 1% of β-TCP content (Figure 12). WCA reduced from 127.3° to 118° upon the addition of 1% of β-TCP into PLLA/CMS nanofibers, which was reduced by about 11%. Ma et al. [58] found WCA had reduced by about 10% for the changes in β-TCP particles from 10 to 20 wt % in PLLA. A similar observation was reported by Yang et al. [59] in TiO_2_/PMMA composite film. 

A small change in WCA of PLLA/CMS/β-TCP was related to the existence of a high porosity that caused macroscopic roughness at the surface. Changes in surface macroscopic level at different scales impede the improvement to a hydrophilic surface. The β-TCP particles in nanoscale also contribute to the increase in the surface roughness of the composite nanofibers, thus reducing the hydrophilic properties.

### 3.6. Mechanical Strength

**Figure 13:**
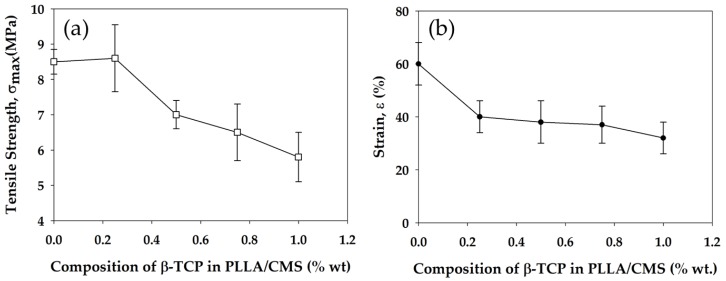
Changes in tensile strength (**a**) and strain (**b**) of PLLA/CMS/β-TCP composite nanofibers with the increase of the β-TCP content in the composition.

Mechanical strength of the composite nanofibers was measured by the tensile strength test. Generally, the mechanical strength of composite nanofibers decrease with the increase in the β-TCP concentration. There was a slight increase in tensile strength at 0.25% of β-TCP concentration and a decrease from 8.5 down to 5.7 MPa after increasing the content to up to 1%. At lower content, the β-TCP acts as a barrier in molecular movement during the tensile test which results in slight increase (Figure 13a). The weakness in tensile strength with the increase in the β-TCP content can be related to the disruption at both the natural and synthetic polymer structure by the particles. The disruption causes stress concentration at composite nanofibers. The non-uniform particle distribution tends to agglomerate, which damages the structure of individual fibers. Fundamentally, tensile strength has a great relation with the critical defect size in material structure. Weakness in interface bonding between β-TCP and PLLA/CMS led to the weaker tensile strength. Defects and porosity arising from the particles also contributed to the reduction in tensile strength [60].

Several observations have reported the relationship of a decrease in tensile strength in nanofibers to the presence of particles in the polymeric structure of either HA or TCP particles [42,61]. However, there are a number of studies which also reported an increase in tensile strength with regards to an addition of the particles to polymers nanofibers [62,63,64]. Strain properties of the composite nanofibers also show a decrease with the increase of the β-TCP particles (Figure 13b). Generally, the failure in materials is due to two fundamental reasons—either the maximum strength has reached the level related to the stiffness of the materials, or the existence of stress concentration in the structure. In our case, stress concentration due to particles in the polymeric structure contributed to the rapid failure in the composite nanofibers. The reduction in crystallization in the polymeric region was also caused by the reduction in strain.

## 4. Conclusions

In this study, we prepared and optimized a composite of synthetic–natural–inorganic mineral nanofibers with the potential to be applied as biomedical membranes, such as guided bone regeneration (GBR) membranes. The composite nanofiber mat was successfully electrospun to uniform non-woven nanofibers. The combination of natural polymers and β-TCP mineral improved the hydrophilic behavior of the PLLA but a higher concentration of the β-TCP led to decreases in mechanical strength and an increment in fiber size. Agglomeration may restrain the performance of the uniform and smooth nanofibers. In comparison to the properties of other research works in the area of nanofibers that have undergone cell studies, overall, the engineered electrospun nanofibers prepared in this works are promising candidates for bone tissue engineering applications such as the GBR membrane. Based on the presented work, this research topic has a great potential to be further investigated for degradation behavior, cell and materials interactions, and in vivo studies towards the bone tissue engineering applications. 

## Figures and Tables

**Table 1:** Data from the DSC curves for the changes in the β-TCP content in the composite nanofibers.

Samples	*T*_g_ (°C)	*T*_c_ (°C)	*T*_m_ (°C)	* ∆*H*_c_ (J/g)	* ∆*H*_m_ (J/g)	* *X*_c_ (%)
PLLA/CMS	53.6	69.0	170.0	9.5	45.8	38.2
0.25% β-TCP	56.6	70.8	171.7	8.7	42.3	35.3
1 % β-TCP	55.0	67.5	166.1	14.1	35.0	22.0

* ∆*H*_c_ and ∆*H*_m:_ Cold Crystallization and Melting Enthalpy, * *X*_c_: Degree Crystallization.

**Table 2:** In crystallite size with the increasing concentration of the β-TCP in the composition.

Sample (%β-TCP)	Peaks Intensity (cps)	FWHM (radian)	Crystallite Size (nm)
0 0.25 0.5 0.75 1.0	138.5 57.4 45.9 37.2 106.8	0.050 0.0261 0.0346 0.0376 0.0510	0.506 0.976 0.736 0.692 No data obtained
